# Global transcriptional response to mammalian temperature provides new insight into *Francisella tularensis *pathogenesis

**DOI:** 10.1186/1471-2180-8-172

**Published:** 2008-10-08

**Authors:** Joseph Horzempa, Paul E Carlson, Dawn M O'Dee, Robert MQ Shanks, Gerard J Nau

**Affiliations:** 1 Department of Microbiology and Molecular Genetics, University of Pittsburgh School of Medicine, Pittsburgh, PA 15261, USA; 2 Charles T. Campbell Laboratory of Ophthalmic Microbiology, Department of Ophthalmology, University of Pittsburgh Eye Center, Pittsburgh, PA 15213, USA; 3 Department of Medicine, Division of Infectious Diseases, University of Pittsburgh School of Medicine, Pittsburgh, PA 15261, USA; 4 Center for Vaccine Research, University of Pittsburgh School of Medicine, Pittsburgh, PA 15261, USA

## Abstract

**Background:**

After infecting a mammalian host, the facultative intracellular bacterium, *Francisella tularensis*, encounters an elevated environmental temperature. We hypothesized that this temperature change may regulate genes essential for infection.

**Results:**

Microarray analysis of *F. tularensis *LVS shifted from 26°C (environmental) to 37°C (mammalian) showed ~11% of this bacterium's genes were differentially-regulated. Importantly, 40% of the protein-coding genes that were induced at 37°C have been previously implicated in virulence or intracellular growth of *Francisella *in other studies, associating the bacterial response to this temperature shift with pathogenesis. Forty-four percent of the genes induced at 37°C encode proteins of unknown function, suggesting novel *Francisella *virulence traits are regulated by mammalian temperature. To explore this possibility, we generated two mutants of loci induced at 37°C [FTL_1581 and FTL_1664 (*deoB*)]. The FTL_1581 mutant was attenuated in a chicken embryo infection model, which was likely attributable to a defect in survival within macrophages. FTL_1581 encodes a novel hypothetical protein that we suggest naming *t*emperature-*i*nduced, *v*irulence-associated locus *A*, *tivA*. Interestingly, the *deoB *mutant showed diminished entry into mammalian cells compared to wild-type LVS, including primary human macrophages and dendritic cells, the macrophage-like RAW 264.7 line, and non-phagocytic HEK-293 cells. This is the first study identifying a *Francisella *gene that contributes to uptake into both phagocytic and non-phagocytic host cells.

**Conclusion:**

Our results provide new insight into mechanisms of *Francisella *virulence regulation and pathogenesis. *F. tularensis *LVS undergoes considerable gene expression changes in response to mammalian body temperature. This temperature shift is important for the regulation of genes that are critical for the pathogenesis of *Francisella*. Importantly, the compilation of temperature-regulated genes also defines a rich collection of novel candidate virulence determinants, including *tivA *(FTL_1581). An analysis of *tivA *and *deoB *(FTL_1664) revealed that these genes contribute to intracellular survival and entry into mammalian cells, respectively.

## Background

*Francisella tularensis *is a Gram-negative bacterium that is pathogenic to humans [1]. This organism causes mortality in up to 60% of infected individuals if untreated [2]. Based on the potential to weaponize this organism, the Center for Disease Control and Prevention has classified *F. tularensis *as a Category A biodefense agent [3]. It is therefore vital to understand how this organism responds to environmental and host signals, and how these cues alter expression of virulence determinants. During the course of a natural *Francisella *infection, this bacterium may transition from an amoeba [4] or an arthropod host [5] to colonize human cells. Accompanying this transition, it is likely that chemical and physical signals alert *Francisella *that it has entered a mammalian host.

The manner by which *F. tularensis *integrates environmental stimuli to regulate gene expression is fundamental to the success of this organism as an intracellular pathogen. The most well-studied virulence factors of *F. tularensis *are encoded in the *Francisella *Pathogenicity Island (FPI) [6]. The amount of one of the virulence proteins encoded in this cluster, IglC, increases in response to growth in macrophages and hydrogen peroxide [7]. In addition, iron limitation has been shown to induce transcription and protein levels of IglACD and PdpB [8,9] as well as stimulate siderophore production by *F. tularensis *[10]. Previously, we have shown that differing culture conditions greatly influence host-pathogen interactions and the ability of *F. tularensis *live vaccine strain (LVS) to activate macrophages [11].

Prior to the current study, there has only been a single published report characterizing the global transcriptional *Francisella *response to an environmental cue, specifically iron limitation [8]. Important insights into the regulation of virulence factors like *iglC *were defined in this analysis. However, numerous genes associated with virulence by other studies are not affected by different iron concentrations [8,12-15]. Because *F. *tularensis may transition between hosts, mammalian body temperature is another signal that is likely to be critical for this pathogen.

Pathogenic bacteria that encounter a shift in temperature during their life cycle sometimes respond with enhanced virulence factor expression [16-19]. However, there are discrepancies among the specific groups of genes that are affected by temperature and the mechanism of regulation between organisms. For example, *Shigella *increases production of its Type III secretion system in response to mammalian temperature [20]. The homologous secretion apparatus in pathogenic *Salmonella*, however, is not regulated by temperature [21]. Regarding the mechanism of regulation, genes involved in the heat-shock response are often induced at mammalian temperatures relative to those of the environment. This regulation is usually due to the presence of a conserved inverted repeat regulatory structure in the promoter region [22], or control by a σ^32^-type heat shock sigma factor [23]. In contrast, the bacterium responsible for whooping cough, *Bordetella pertussis*, uses a two-component system comprised of BvgS and BvgA to alter transcription of genes in response to temperature. Following induction of *bvgAS *at 37°C, phosphorylation by BvgS allows BvgA-binding to promoter regions of virulence genes, such as the adhesin, *fimX *[24]. Given the uncertainty of target temperature-regulated genes and differences in mechanism among bacteria, it is necessary to define the temperature regulon in individual species. Studying gene expression changes induced by a shift to mammalian temperature could provide valuable insight into *Francisella *virulence.

A few previous studies have investigated the *Francisella *response to temperature at the molecular level. High temperature (42–44°C) synonymous with heat stress has been shown to have dramatic effects on *F. tularensis *gene and protein expression [25-27] including increased production of the heat shock proteins GroEL, GroES, DnaK, and ClpB [27]. Heat stress has also been shown to enhance the virulence of a mutant form of *F. tularensis *LVS [28]. Interestingly, *F. novicida *has been shown to alter its outer membrane at 25°C versus 37°C by differentially modifying the lipid A component of the lipopolysaccharide [29]. In addition, it has been observed that virulent *F. tularensis *clinical isolates increase the mannose modification of their lipopolysaccharide when cultivated at temperatures less than 25°C [30]. The results from these previous studies suggest that a global transcript analysis following a shift to mammalian body temperature should reveal gene regulation that is critical to *Francisella *pathogenesis.

Here we show that a shift from environmental to mammalian body temperature significantly alters the transcriptome of *F. tularensis *LVS. Many genes that we identified as significantly induced at 37°C have been previously implicated in *Francisella *virulence, supporting the notion that this temperature shift is important for the regulation of pathogenesis. We provide evidence that the product of a gene encoding the hypothetical protein most profoundly induced by mammalian temperature contributes to virulence and intracellular growth. Moreover, data presented here indicate that a locus up-regulated at 37°C was required for optimal uptake by eukaryotic cells. This is the first *F. tularensis *gene identified to be involved in the entry of this organism into both phagocytic and non-phagocytic host cells. The results from this study support a model where detection of mammalian body temperature by *F. tularensis *is important for regulation of physiology necessary for successful infection.

## Results

### *F. tularensis *LVS global temperature regulation

**Figure 1:**
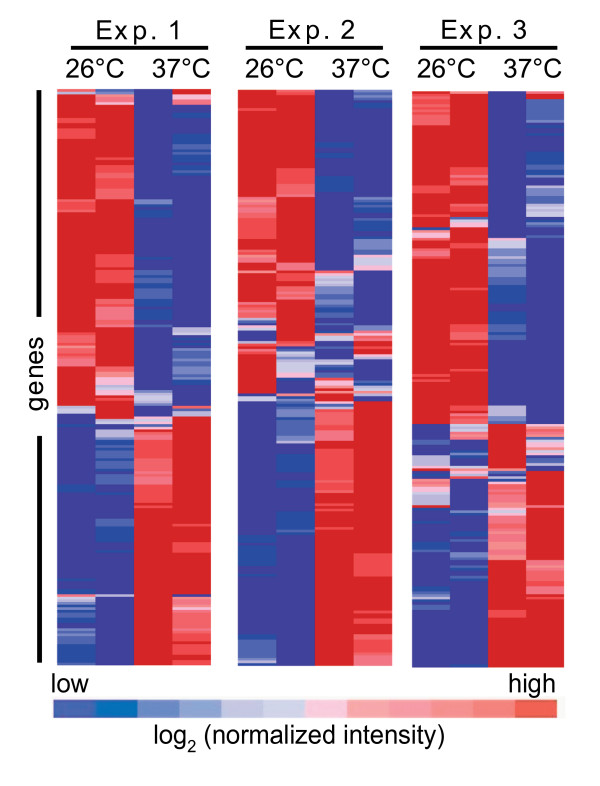
**Global *Francisella *gene expression changes induced by mammalian body temperature**. Genes that were identified as statistically significant by J5 scores across three independent microarray experiments were subjected to hierarchical clustering. Fluorescence intensities were standardized by the minimum mean ratio array normalization followed by log_2 _transformation. Values were clustered using the Pearson correlation in GenePattern by independently inputting individual data from each experiment. Oligonucleotides testing individual LVS ORFs are in duplicate on each array and the results are displayed as two columns per experiment in GenePattern. The 95 induced genes and 125 repressed genes clustered together in this GenePattern output, validating the J5 statistical analysis from GEDA. (B).

To study *Francisella *gene expression changes associated with exposure to mammalian temperature, we conducted a microarray analysis of LVS as a model *F. tularensis *strain (Table 1). Gene expression of LVS cultured at 26°C (non-mammalian environment) was compared to bacteria shifted to 37°C (mammalian host body temperature). Labeled cDNA target was produced from RNA isolated from *F. tularensis *LVS and was subsequently hybridized to a custom Agilent *Francisella *microarray. Global gene expression data were analyzed with a J5 statistical test, which was selected to limit the number of false positives [31]. This analysis identified 95 genes with significantly increased expression and 125 genes with decreased expression in response to a shift to mammalian body temperature (see additional files 1 and 2, respectively). Collectively, this represents approximately ~11% of the genes in the entire LVS genome. Genes with a significant change in expression were examined with the Gene Pattern program by hierarchical clustering (Fig. 1). The clustered data exhibited a distinct pattern of transcript induction and repression in response to mammalian body temperature (Fig. 1). Together, these data indicate that this temperature shift has a broad impact on *F. tularensis *transcription.

**Table 1:** Strains, plasmids, and primers used in this study.

Strain, plasmid, or primer	Description	Source or Reference
**Strains**		
*F. tularensis*		
LVS	*F. tularensis *subsp. *holartica *live vaccine strain	Karen Elkins
1581d	LVS with *FTL_1581 *disruption, Hyg^R^	This study
1664d	LVS with *FTL_1664 *(*deoB*) disruption, Hyg^R^	This study
		
*E. coli*		
XL10-Gold	Δ(*mcrA*)*183 Δ *(*mcrCB-hsdSMR-mrr*)*173 endA1 supE44 thi-1 recA1 gyrA96 relA1 lac *Hte [F' *proAB lacI*^q^*ZDM15 Tn10 *(Tet^R^) Amy Cam^R^]	Stratagene
DH5α	F-φ80*lac*ZΔM15 Δ(*lac*ZYA-*arg*F) U169 *rec*A1 *end*A1 *hsd*R17 (r_k_-, m_k_+) *pho*A *sup*E44 λ-*thi*-1 *gyr*A96 *rel*A1	Invitrogen
JM109	*end*A1, *rec*A1, *gyr*A96, *thi, hsd*R17 (r_k_^-^, m_k_^+^), *rel*A1, *sup*E44, Δ(*lac-pro*AB), [F' *tra*D36, *pro*AB, *laq*I^q^ZΔM15]	Promega
		
**Plasmids**		
pFNLTP8	*Francisella *shuttle plasmid, Ap^R^, Km^R^	[63]
pRK2013	Helper plasmid for triparental mating, Km^R^	[64]
pMP615	*Francisella *shuttle plasmid, Hyg^R^	[65]
pMQ131hyg	*F. tularensis *suicide vector, pBBR1 *ori*, *oriT*, contains the *hyg *cassette driven by the *groEL *promoter from pMP615, *oriT*, Km^R^, Hyg^R^	This study
pMQ131hyg1581d	pMQ131hyg with the central 560 base pair region of FTL_1581	This study
pMQ131hyg1664d	pMQ131hyg with the central 900 base pair region of FTL_1664 (*deoB*)	This study
pFTL_1581	Broad-host-range vector, pC194 *ori*, contains cloned FTL_1581 along with 600 base pairs upstream and 100 base pairs downstream of this gene, complementing plasmid, Ap^R^, Cam^R^	This study
pF8AX	pFNLTP8 Δ*npt*, Ap^R^	This study
pF8CAT	pF8AX with *cat*, Ap^R^, Cam^R^	This study
pFTL_1664	pF8CAT with cloned FTL_1664 (*deoB*) along with 600 base pairs upstream and 100 base pairs downstream of this gene, complementing plasmid	This study
		
**Primers**		
1581_560F	5'-ATGGATCCTGAGCTAAATGATGCTTTAGTATCTC-3'	Invitrogen
1581_560R	5'-ATGGTACCAAGACGACATAGCCACG-3'	Invitrogen
1664_900F	5'-ATGGATCCGAACCTGGAGCAGTTGAAT-3'	Invitrogen
1664_900R	5'-ATGGTACCTAAGAAAGTTGCGGAATATAATAGATG-3'	Invitrogen
1581_clone_up	5'-GATCGGATCCAGGTCAATCAGGAGTTGG-3'	Invitrogen
1581_clone_down	5'-GATCGGTACCCACCTATTTGAATTAAAAAGAAGTTTATACAC-3'	Invitrogen
1664_clone_up	5'-GATCGGATCCGATGGCTATGGTATATCTTCGG-3'	Invitrogen
1664_clone_down	5'-GATCGGTACCACCGAGAGAATTTCTCGC-3'	Invitrogen
F8AgeI	5'-CATTAGACCGGTGCGAAACGATCCTCATCCTGTC-3'	Invitrogen
F8XhoI	5'-Phosphorylated – CATTAGCTCGAGGGAAGAGTATGAGTATTCAAC-3'	Invitrogen
XhoICAT	5'-CATGCTCGAGTTATAAAAGCCAGTCATTAGGCC-3'	Invitrogen
AgeICAT	5'-CATGACCGGTATGAACTTTAATAAAATTGATTTAGACAATTGG-3'	Invitrogen

**Figure 2:**
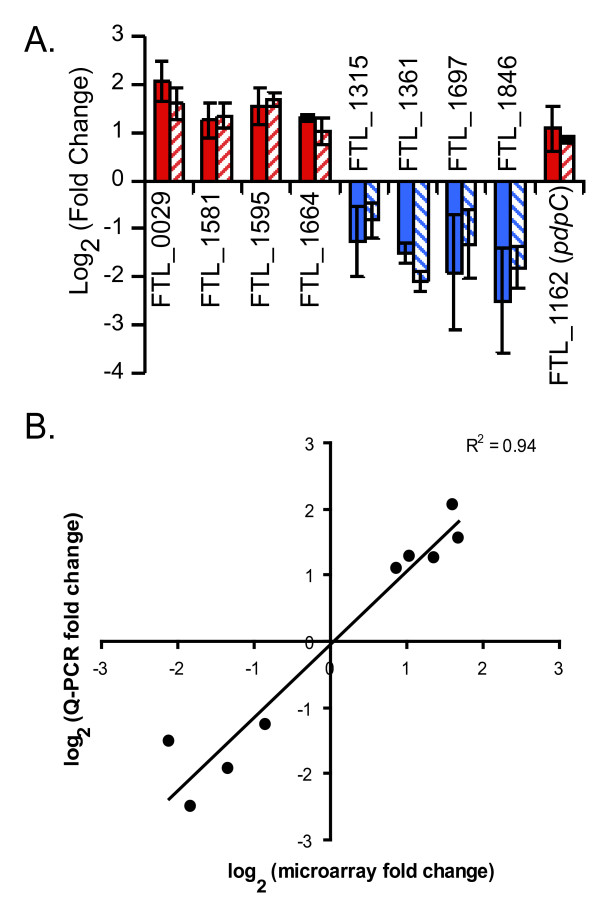
**Validation of the microarray results and comparison with Q-PCR**. (A) RNA from the microarray experiments depicted in panel A were tested for up- or down-regulation of specific genes using Q-PCR. Q-PCR data are represented with solid bars, whereas values from microarray experiments are depicted as striped bars. Both data sets are presented as mean ± SEM from three individual experiments. (B) Correlation analysis of the microarray and Q-PCR transcript measurements for nine select *F. tularensis *LVS ORFs. The microarray log_2 _values were plotted against the Q-PCR log_2 _data. The correlation coefficient (R^2^) between the two analyses is 0.94.

The microarray data were confirmed using quantitative real time PCR (Q-PCR), in which eight differentially-expressed genes were analyzed, representing transcripts that were both significantly up- and down-regulated (Fig. 2A). Induced genes chosen for validation were the carbamoyl-phosphate synthase large chain, *carB *(FTL_0029), a hypothetical lipoprotein (FTL_1581), a dimethyladenosine transferase annotated to function in kasugamycin resistance, *ksgA *(FTL_1595), and phosphopentomutase, *deoB *(FTL_1664). The down-regulated genes tested by Q-PCR included two hypothetical proteins (FTL_1315 and FTL_1846), a cold shock protein, *cspC *(FTL_1361), and a metal ion transporter, *tlyC *(FTL_1697). A similar pattern of expression was observed in both Q-PCR and in the microarray experiments (Fig. 2A). A correlation plot (Fig. 2B) showed a strong, positive association between both data sets (R^2 ^= 0.94). This indicated the microarray platform and the subsequent statistical analysis were robust compared to the sensitive, though low-throughput Q-PCR approach.

**Figure 3:**
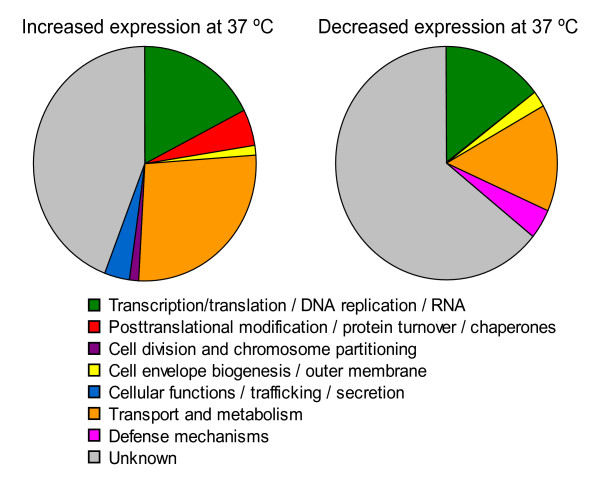
**COG analysis of genes up- or down-regulated by mammalian temperature**. COG categories were identified for each significantly up- or down-regulated gene based on the genomic annotation (accession, NC_07880). Specific COG categories were consolidated into general categories as follows: J, K, L, and RNA genes were combined into "Transcription/translation/DNA replication/RNA"; P, C, G, E, F, H, I, and Q were merged into "Transport and metabolism"; categories N and U were combined into "Cellular functions/trafficking/secretion"; and categories R, S, and uncategorized genes were classified as "Unknown". All other COG categories were reported as they were originally assigned.

To analyze the differentially-regulated genes more thoroughly, we categorized their presumed protein products based on their Clusters of Orthologous Groups (COG) category. As expected, many of the induced and repressed genes were involved in central biological functions such as metabolism, transcription, translation, DNA replication, and RNA genes (Fig. 3). Heat shock proteins belong to the category labeled "posttranslational modification/protein turnover/chaperones", and as anticipated, these genes were induced at the higher temperature, providing additional support to our microarray analysis (Fig. 3). The heat shock protein result was confirmed by analyzing Hsp70 protein quantity by Western blotting, which showed significantly more Hsp70 in bacteria shifted to 37°C versus *F. tularensis *LVS cultured at 26°C (data not shown). COG categories found only among the up-regulated transcripts included genes functioning in secretion and cell division, suggesting that host temperature may trigger these cellular processes and reflect, in part, enhanced growth rate (Fig. 3). In addition, the COG for bacterial defense mechanisms, which includes type I site-specific restriction-modification systems, was uniquely down-regulated. This response may have evolved to allow this bacterium to better contend with environmental stresses, such as invading bacteriophages. We observed that a large percentage of both up- and down-regulated genes (44% and 64% respectively) did not belong to a known COG category (Fig. 3). Proteins with an unknown COG category may have a novel biological role in *F. tularensis *associated with their temperature regulation.

### *F. tularensis *LVS temperature regulation of genes necessary for infection

We hypothesized that the shift from environmental to mammalian temperature may be used to regulate genes important for infection. Surprisingly, none of the genes encoded in the FPI were significantly up-regulated at 37°C in the microarray analysis. We also confirmed, by immunoblotting, that IglC protein levels were equivalent in bacteria grown at the two different temperatures (data not shown). Upon further scrutiny, we did notice that one gene in the FPI, *pdpC *(FTL_0116; FTL_1162), was near the statistical threshold for induction at 37°C. We therefore analyzed *pdpC *transcript levels by Q-PCR as before, which confirmed that this gene was induced at mammalian body temperature (Fig. 2A).

We next compared our list of genes induced at 37°C with those previously shown to be necessary for *Francisella *infection, or postulated to be involved in the pathogenesis of this organism (Table 2). Forty percent of the protein-coding genes significantly up-regulated at 37°C have been reported or predicted to be important for intracellular growth and/or virulence of *F. tularensis *(Table 2). The list depicted in Table 2 included an assortment of metabolic genes, chaperones, genes encoding hypothetical proteins, and others. The data from Table 2 and the *pdpC *expression results in Fig. 2A further supported our hypothesis that genes important for *Francisella *infection are regulated by mammalian body temperature.

**Table 2:** List of genes induced at 37°C shown or implicated to be associated with intracellular growth and/or virulence of *F. tularensis*.

Locus tag	Description, gene name (if available)	Fold Change	Reference
FTL_0028	Aspartate carbamoyltransferase, *pyrB*	2.2	[12,14]
FTL_0029	Carbamoyl-phosphate synthase large chain, *carB*	2.6	[12,14,66]
FTL_0030	Carbamoyl-phosphate synthase small chain, *carA*	2.6	[12,14]
FTL_0094	Chaperone, *clpB*	2.5	[13,14,27,67,68]
FTL_0198	Pyridoxal/pyridoxine/pyridoxamine kinase, *pdxY*	2.0	[14]
FTL_0267	Chaperone Hsp90, *htpG*	2.3	[14,46,66,69]
FTL_0307	Dephospho-CoA kinase, *coaE*		[46,69]
FTL_0337	Pseudogene with homology to *miaB*	2.1	[13]
FTL_0445	Hypothetical protein with homology to NADPH-dependent FMN reductase	2.5	[13,46,69]
FTL_0479	glycine cleavage system P protein, subunit 1, *qcvP1*	1.4	[14]
FTL_0671	Annotated as transcriptional regulator, homologous to Pantothenate kinase type III, *coaX*	2.3	[46,69]
FTL_0672	Aspartate-1-decarboxylase, *panD*	2.0	[14,46,69]
FTL_0675	Conserved hypothetical protein	2.3	[46,69]
FTL_0837	D-methionine binding transport protein, ABC transporter, membrane and periplasmic protein, *metlQ*	2.0	[13]
FTL_0885	PhoH-like protein	1.9	[14]
FTL_0886	Conserved hypothetical protein, *yleA*	1.8	[68]
FTL_0899	protease, GTP-binding subunit, *hflX*	1.8	[13]
FTL_0928	DJ-1/PfpI family protein	2.1	[13]
FTL_1048	Conserved hypothetical protein	1.8	[14]
FTL_1190	Chaperone protein (heat shock protein family 70 cofactor), *grpE*	1.7	[46,69]
FTL_1338	Alanine racemase, *alr*	2.1	[67]
FTL_1474	transcriptional elongation factor, *qreA*	1.8	[13]
FTL_1485	Conserved hypothetical membrane protein	2.2	[46,69]
FTL_1545	SNO glutamine amidotransferase family protein	2.6	[46,69]
FTL_1546	Pyridoxine/pyridoxal 5-phosphate biosynthesis protein	2.2	[46,69]
FTL_1553	Succinyl-CoA synthetase beta chain, *sucC*	1.6	[13]
FTL_1595	Dimethyladenosine transferase, kasugamycin resistance, *ksgA*	3.2	[14]
FTL_1664	Phosphopentomutase, *deoB*	2.1	[14]
FTL_1714	Chaperonin (Hsp60 family), *groEL*	3.2	[14]
FTL_1782	adenine phosphoribosyltransferase, *apt*	1.5	[14]

### FTL_1581 is associated with *F. tularensis *LVS virulence

**Figure 4:**
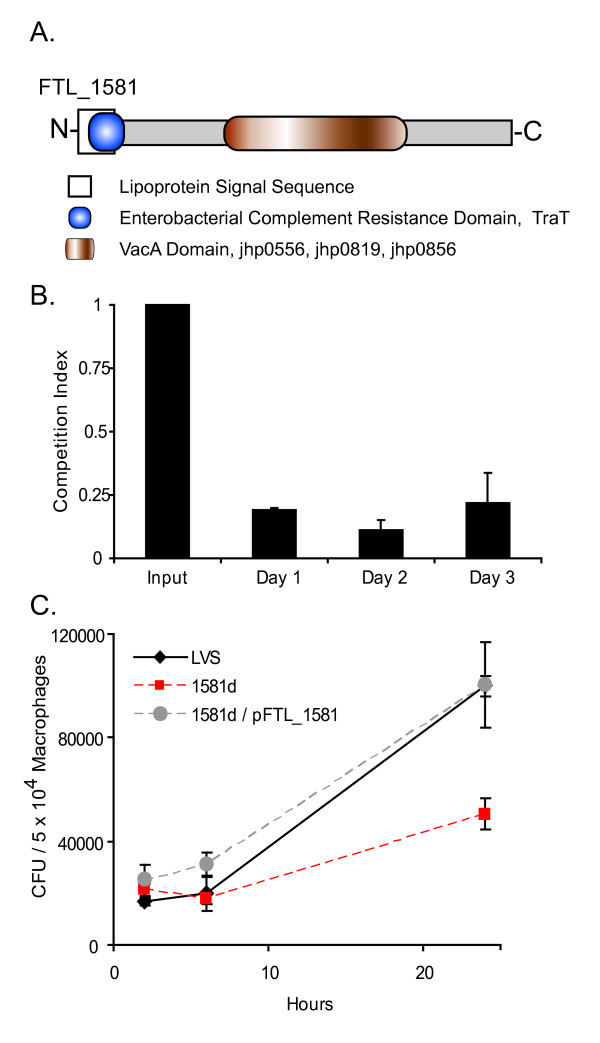
**FTL_1581 contributes to *F. tularensis *LVS virulence**. (A) A schematic of FTL_1581 protein is depicted showing domains predicted by PROSITE. (B) Competition studies in the chicken embryo infection model using a mixture of LVS and 1581d (1:1 based on OD_600_; viable bacteria in the inoculum were quantified by diluting and plating the input). Competition ratios (1581d: LVS) were normalized to the input to account for differences in the inoculum. These ratios were analyzed for statistical significance by Chi square; *P *< 0.001 at days 1, 2, and 3 post-infection. Data are mean competition ratio ± SEM of three embryos per time point within one experiment and are representative of duplicate experiments. (C) Macrophages were infected with LVS, 1581d, or 1581d/pFTL_1581 and were lysed at the indicated times. Data are mean ± SEM of triplicate wells within one experiment and are representative of four experiments performed using cells from separate donors. Following log transformation, differences in CFU were determined by a Student's t-Test in which *P *= 0.003 at 24 h.

FTL_1581, annotated as a hypothetical lipoprotein, was induced by mammalian temperature more profoundly than any other hypothetical protein gene in the *F. tularensis *LVS genome (based on both fold change and J5 score; Additional file 1). We selected this gene for further study to test our hypothesis that temperature regulates genes necessary for infection. A BLAST search [32] against the National Center for Biotechnology Information database of non-redundant sequences did not identify proteins from organisms other than *Francisella *that had >30% identity to FTL_1581 (data not shown). This suggested that FTL_1581 may have a function unique to *F. tularensis*. The primary structure of FTL_1581 was further examined by a PROSITE analysis [33] which indicated this protein likely contained a lipoprotein signal sequence (Fig. 4A) consistent with its annotation. This analysis also revealed that the signal sequence overlapped with a motif similar to that of the Enterobacterial TraT complement resistance protein (Fig. 4A). Although the initial BLAST search did not retrieve homologous proteins with high degrees of identity, it did show that FTL_1581 contained a domain with 20–24% identity and 40–44% similarity with the vacuolating cytotoxin (VacA; jhp0819) [34,35] and paralogs (jhp0556, jhp0856) of *Helicobacter pylori *J99 (Fig. 4A). Because FTL_1581 contained domains similar to other proteins that had a role in pathogenesis, we hypothesized that this protein may contribute to the virulence of *F. tularensis*.

We disrupted FTL_1581 in LVS, producing strain 1581d, to determine if this gene was associated with *F. tularensis *virulence. To assess the virulence of 1581d, we utilized a competition assay based on the chicken embryo infection model [36-38]. The chicken embryo produces a robust innate immune response comprised of complement, phagocytic cells, and cytokine production [39,40]. Here, chicken embryos were infected with a ~1:1 mixture of LVS and 1581d. At days 1, 2, and 3 post-infection, wild-type LVS exhibited superior survival compared to 1581d (*P *< 0.001 at each time point) (Fig. 4B). This result was not due to a growth defect since 1581d grew identically to wild-type LVS when cultivated in bacterial growth medium (data not shown). In a separate experiment in which chicken embryos were infected only with 1581d, isolates from homogenates were all resistant to hygromycin, indicating that this mutant did not revert to wild type during infection (data not shown). Since 1581d was attenuated compared to LVS, the results suggest the function of the FTL_1581 gene product contributes to *F. tularensis *virulence.

We wanted to determine if the virulence attenuation of 1581d in the chicken embryo infection model was due to a reduced ability to inactivate complement, as FTL_1581 contained a putative complement resistance domain (Fig. 4A). Therefore, we subjected LVS and 1581d to serum sensitivity assays. There was equivalent survival of wild-type LVS and 1581d when cultured in media containing 20% serum or 20% heat-inactivated serum for up to 20 h (data not shown). To ensure that the serum complement was functional, *E. coli *DH5α was used as a control. Here, the CFU from the serum-treated *E. coli *exhibited a reduction of 3 logs relative to the input or heat-inactivated serum groups after 30 min (data not shown). This suggested that the disruption in 1581d does not affect complement resistance.

We determined if FTL_1581 contributed to growth in a macrophage environment. Primary human monocyte-derived macrophages were infected *in vitro *with either LVS or 1581d. At various time-points, macrophages were lysed, and the lysates were diluted and plated to enumerate viable CFU. We observed attenuated growth of 1581d in macrophages at 24 h post-infection (*P *= 0.003) (Fig. 4C). When FTL_1581 was complemented *in trans *in 1581d, wild-type level of growth was restored (Fig. 4C). This complementation confirmed that the reduced intracellular fitness of 1581d was due to the inactivation of FTL_1581, and not due to polar effects or alternate mutations. This experiment (Fig. 4C) also suggested that the attenuation of 1581d in the chicken embryo model (Fig. 4B) was likely due to a defect in intracellular survival. Together, the data presented here implicate FTL_1581, a *Francisella *ORF induced by mammalian body temperature that has no obvious homologs, with virulence and intracellular survival. Therefore, we propose that FTL_1581 be named *t*emperature-*i*nduced, *v*irulence-associated locus *A*, or *tivA*.

### Involvement of FTL_1664 (*deoB*) in uptake of *F. tularensis *LVS

Many of the genes critical for *Francisella *infection involved metabolism (Table 2), a major COG category induced at 37°C (Fig. 3). This suggested that physiology vital for the success of *F. tularensis *as a pathogen was regulated by the temperature shift. Therefore, we were interested in determining the contribution of temperature-regulated metabolic genes toward *Francisella *pathogenesis. Previously, a microarray-based negative selection screen of *F. novicida *transposon mutants identified the phosphopentomutase, *deoB*, as a gene contributing to growth and/or survival in mice [14]. In other bacteria, proteins encoded by *deoB *homologs normally catalyze the reversible reaction between ribose-1-phosphate and ribose-5-phosphate or between deoxyribose-1-phosphate and deoxyribose-5-phosphate [41,42]. An *F. tularensis *LVS chromosomal disruption mutant of *deoB*, FTL_1664, was constructed (strain 1664d). This mutant had a cellular and colony morphology similar to its wild-type parent strain (data not shown). Also, 1664d grew similarly to wild-type LVS in bacterial culture medium (data not shown).

We employed the chicken embryo infection model [36-38] to confirm that *deoB *contributes to LVS pathogenesis, as it did in *F. novicida *[14]. Here, chicken embryos that had been infected with *F. tularensis *LVS 1664d exhibited significantly enhanced survival compared to those infected with wild-type LVS over a 5 day period (*P *= 0.0254) (data not shown) corroborating the *deoB *data from *F. novicida *[14].

**Figure 5:**
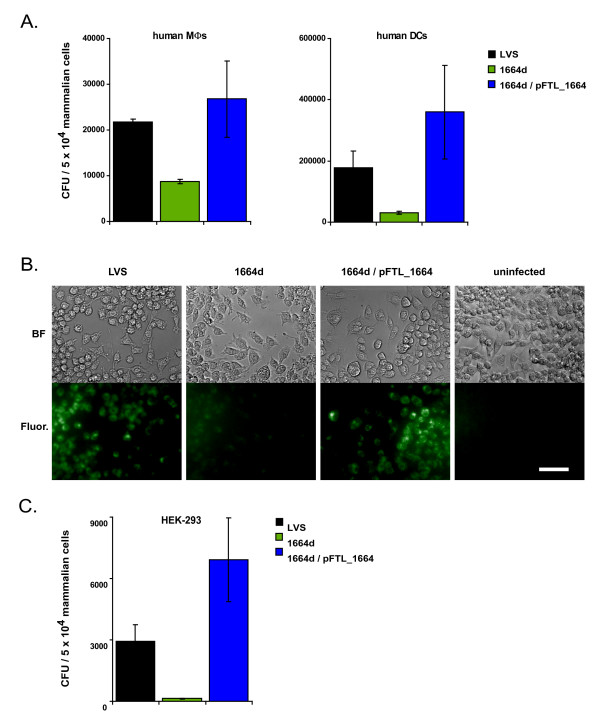
***deoB *is important for entry of *F. tularensis *LVS**. (A) Primary human macrophages (MΦs) or dendritic cells (DCs) were infected with either LVS, 1664d, or 1664d/pFTL_1664 in microtitre plates. After a two-hour incubation, wells were treated with gentamicin to kill extracellular bacteria, followed by vigorous washing. Subsequently, phagocytic cells were lysed and serial dilutions of lysates were plated for CFU enumeration. Data are mean ± SEM of triplicate wells within one experiment and are representative of four (primary human macrophages) or two experiments (dendritic cells) performed using cells from separate donors. Following log transformation, differences in CFU between LVS and 1664d were determined by a Student's t-Test in which *P *= 0.00005 and 0.003 for MΦs and DCs, respectively. (B) Bacteria were stained with the fluorescent green stain, Syto-9 prior to infection. After a two-hour incubation with RAW 264.7 cells, extracellular bacteria were washed away and cells were analyzed under brightfield (BF) and fluorescence (Fluor.) microscopy. The exposure time was extended to enhance sensitivity of detecting the low numbers of 1664d within the RAW 264.7 cells, leading to some background fluorescence. Fluorescence images were initially captured in grayscale and pseudocolored using Adobe Photoshop. Data displayed are representative of duplicate experiments. Scale bar = 50 μm. (C) Human embryonic kidney 293 (HEK-293) cells were infected similarly to the phagocytic cells in panel A. Data are mean ± SEM of triplicate wells within one experiment and are representative of three experiments. Following log transformation, differences in CFU between LVS and 1664d were determined by a Student's t-Test in which *P *= 0.0004.

Accessing the host cytoplasm to replicate intracellularly is a hallmark of *Francisella *pathogenesis. Therefore, we next tested if the virulence attenuation of 1664d in the chicken embryo infection model was due to a defect in entering host cells. At two hours post-infection *in vitro*, human, monocyte-derived macrophages were treated with gentamicin to kill extracellular bacteria, followed by extensive washing. We consistently observed that substantially fewer 1664d cells were phagocytosed relative to wild-type LVS (Fig. 5A) (*P *= 0.00005). Importantly, *trans *complementation of 1664d (1664d/pFTL_1664) rescued the uptake defect (Fig. 5A). This suggested *deoB *is involved in a bacterial mechanism that enhances uptake of *F. tularensis*. This finding was extended by conducting similar uptake assays in primary human dendritic cells (Fig. 5A). In both phagocytic cell types, the uptake defect of 1664d was reproduced and complemented (Fig. 5A).

We then qualitatively assessed uptake into phagocytes by microscopy to confirm the quantitative results obtained with CFU measurements. LVS, 1664d, and 1664d/pFTL_1664 were treated with the green fluorescent stain, Syto-9, and then incubated with RAW 264.7 cells, a murine macrophage-like cell line. After a two hour incubation and washes, cells were observed by fluorescence microscopy. Here, RAW 264.7 cells infected with LVS or 1664d/pFTL_1664 exhibited bright green fluorescence (Fig. 5B). In contrast, the RAW 264.7 cells infected with 1664d produced considerably less fluorescence despite comparable inocula to the cultures (Fig. 5B). The results obtained by microscopy suggested the discrepancy in CFU between wildtype and 1664d was not due to killing in the early phagosome. Rather, the levels of fluorescence in Fig. 5B were consistent with the CFU uptake data presented in Fig. 5A and confirmed that the temperature-regulated *deoB *is important for optimal uptake by mammalian phagocytes.

To determine if *deoB *contributed to entry into non-phagocytic cells, we exposed the human embryonic kidney cell line HEK-293 to either LVS, 1664d, or 1664d/pFTL_1664. At two hours post-infection, these cells were treated with gentamicin to kill extracellular bacteria followed by extensive washing. Subsequently, HEK-293 cells were lysed and the lysates were diluted and plated to enumerate CFU. Here we observed that 1664d showed reduced entry into the non-phagocytic HEK-293 cells (Fig. 5C). The uptake defect was again rescued by *trans *complementation (Fig. 5C). These data suggest that *deoB *contributes significantly to entry into both phagocytic (Fig. 5A and 5B) and non-phagocytic cells (Fig. 5C).

## Discussion

In this paper, we provide evidence that *F. tularensis *LVS undergoes significant gene expression changes in response to mammalian body temperature (Fig. 1 and 2, Table 2, and Additional files 1 and 2). We hypothesized that genes important for infection of mammals may be induced during transition to this higher temperature. Although some genes, such as ribosomal RNA and tRNA genes, may reflect an increased growth rate at 37°C, our overall hypothesis was supported by the finding that 40% of the protein coding genes induced at 37°C have been shown or implicated to be important for successful *Francisella *infection (Table 2). In addition, we showed that two specific genes induced at 37°C contribute to the fitness of *F. tularensis *LVS during infection. While infection of a mammalian host exerts a complex set of signals on *F. tularensis *in addition to temperature, it is likely that many of the unexplored genes induced at mammalian temperature have a role in pathogenesis of *F. tularensis*. Differential responses to temperature among virulent and less virulent *F. *tularensis strains will also provide a focused list of relevant candidate virulence determinants to investigate further. The products of temperature-regulated genes with central roles in physiology and virulence could be targets for novel therapeutics or mutation to generate defined live attenuated vaccines.

One gene induced at 37°C, *tivA *(FTL_1581), encodes a protein with little overall identity to other proteins. It does, however, contain regions of meager similarity to the domains of proteins involved in the pathogenesis of other bacteria (Fig. 4A). We showed that this gene was necessary for full *F. tularensis *LVS virulence in the chicken embryo model, most likely because it contributed to optimal replication in primary human macrophages (Fig. 4B and 4C). In a previous high-throughput microarray-based, negative-selection screen for *F. novicida *genes important for a murine infection, the *tivA *allele of *F. novicida *(FTN_0573) was not identified [14]. There are two possible explanations for this discrepancy. There may be intrinsic differences between *F. tularensis *LVS and *F. novicida *pathogenesis [43] and their reliance on *tivA*. A more probable explanation is that *F. tularensis *LVS and the more pathogenic *F. tularensis *Schu S4 contain a single copy of this gene. In contrast, *F. novicida *has two homologs of *tivA *in its genome, suggesting the possibility of functional redundancy. These alternate forms (FTN_1103, 52% identity; FTN_1101, 49% identity) may have compensated for a mutation of *tivA *(FTN_0573) during the negative-selection screen [14], thereby masking any effects on pathogenesis. This possibility underscores the value of analyzing several different strains when probing *F. tularensis *pathogenesis.

Strain 1664d, a disruption mutant of *deoB*, exhibited significant reduction in uptake into mammalian cells (Fig. 5) indicating that this gene's product is involved in a mechanism that enhances entry. It would be advantageous for *Francisella *to couple expression of a gene important for uptake (*deoB*) with mammalian temperature, a cue encountered early in infection. Augmenting entry would improve access to the intracellular environment in which *F. tularensis *proliferates [44]. A previous study showed that *pyrB *(FTT1665) from *F. tularensis *Schu S4 was important for invasion in the human HepG2 hepatocellular carcinoma line [12]. However, a Schu S4 mutant of *pyrB *was not defective in uptake by J774.1 macrophage-like cells [12], suggesting that the *Francisella *mechanisms of optimal uptake by phagocytes and invasion into hepatocytes are separate phenomena. In another prior study, mutants of MglA, a key regulator of the FPI [45], and mutants of six genes controlled by this protein were not defective for cell entry [46]. This suggests that *Francisella *has evolved separate regulatory mechanisms for enhancing uptake and for intracellular survival. However, based on our data, genes critical for both phenomena are affected by mammalian temperature indicating that this cue is an important signal for multiple regulatory networks in *Francisella*. The uptake defect we have shown with the *deoB *mutant in both phagocytic and non-phagocytic cells has not been described previously.

In our system, DeoB may have a direct or indirect role in the uptake of *Francisella*. DeoB may influence the LPS structure of *Francisella*, secondarily improving interactions with host cell receptors. Importantly, complement receptors and mannose receptors are crucial for optimal *Francisella *phagocytosis [47-50]. This model is consistent with the fact that *deoB *is induced at 37°C and the finding that *Francisella *LPS structure is different at lower temperature versus mammalian temperature [29,30]. Alternatively, DeoB may directly promote *Francisella *entry into host cells as this protein may have an additional function aside from being a phosphopentomutase. Another possibility is that mutation of metabolic genes in pathogenic bacteria can yield pleiotropic effects, resulting in defects in virulence mechanisms, including invasion [12]. Further investigation is necessary to determine the precise mechanism of *Francisella *DeoB in host cell entry.

The data presented here suggest that some genes important for *Francisella *during infection are induced by mammalian temperature. Although we showed by Q-PCR that *pdpC *transcripts were induced at 37°C, none of the other loci in the FPI [6] were up-regulated at mammalian body temperature. This is consistent with other findings suggesting that IglC protein levels were not induced by a shift from 37°C to 42°C [7]. Because many of the loci in the FPI are essential for intra-amoeba growth [45], which would occur at lower, ambient temperatures, it seems logical that these genes are not regulated by temperature. Therefore, the virulence associated-genes induced by mammalian temperature that we have identified are on a separate regulon than most of the genes of the FPI. This suggests *Francisella *possesses an intricate regulatory circuit to maximize its success in diverse environments.

## Conclusion

*F. tularensis *LVS undergoes significant gene expression changes in response to mammalian temperature. This temperature shift is important for the regulation of pathogenesis. Our study characterizes a previously underappreciated environmental cue that regulates the expression of *F. tularensis *genes associated with virulence in other studies. Importantly, the collection of temperature-regulated genes also defines a rich set of novel candidate virulence determinants, including *tivA *(FTL_1581). Detailed investigation of *tivA *and *deoB *(FTL_1664) revealed unknown or unrecognized roles of these genes in intracellular survival and entry into mammalian cells, respectively.

## Methods

### Bacterial strains and growth conditions

Bacterial strains used in this study can be found in Table 1. All broth cultures were grown with agitation (250 rpm). For general cultivation of *Escherichia coli*, bacteria were grown at 37°C on LB agar plates, or in LB broth. *F. tularensis *LVS, a model organism for tularaemia, was used in this study. For *F. tularensis *LVS strains, frozen stock cultures were streaked onto chocolate II agar plates and incubated at 37°C, 5% CO_2 _for 2–4 days. These bacteria were subsequently used to inoculate broth cultures. For experiments assessing the effect of temperature on transcript levels, Chamberlain's chemically defined broth medium (CDM) [51] was inoculated with LVS and incubated at 37°C overnight. This start-up culture was used to inoculate fresh CDM (2 ml start-up culture into 25 ml fresh broth) and incubated at 26°C for 24 h. Following this incubation, the optical density (OD_600_) of this culture was recorded and RNA was extracted. For the temperature shift, 5 ml of the same culture was diluted into 25 ml fresh CDM and incubated at 37°C until attaining an OD_600 _comparable to the 26°C culture (typically ~6 h). When the 37°C culture reached the desired OD_600_, RNA was harvested. This strategy of growing bacteria to comparable OD_600 _prior to RNA extraction was similar to a previous study assessing global temperature regulation in Group A *Streptococcus *[19] and mitigates the effects of growth phase on the subsequent analysis.

For macrophage and chicken embryo infections, LVS strains were grown in TSBc (trypticase soy broth [Becton, Dickinson and Company] supplemented with 0.1% L-cysteine hydrochloride monohydrate) at 37°C while shaking at 250 rpm. When required, antibiotics were added to the media at the following concentrations: ampicillin at 150 μg/ml for *E. coli*; kanamycin at 35 μg/ml for *E. coli *and 10 μg/ml for *F. tularensis *LVS; chloramphenicol at 5 μg/ml for *F. tularensis *LVS; polymixin at 100 μg/ml; and hygromycin at 200 μg/ml.

The rationale for using CDM for the transcriptional analysis, while TSBc was used for all other experiments is two-fold. Using CDM for general cultivation of LVS is not practical because CDM is an aqueous mixture of 22 nutrients, some of which do not have a long shelf-life. Secondly, CDM was selected for transcriptional analyses to benefit future studies to analyze synergistic effects of temperature and media components.

### RNA extraction

Immediately upon removal from the shaking incubator, six ml of broth culture were mixed with 18 ml TriReagent LS (Molecular Research Center). Chloroform (4.8 ml) was added to this material, and the aqueous phase was subsequently separated by centrifugation in a Phase Lock Heavy tube (Eppendorf). RNA was precipitated from this aqueous layer with isopropanol, followed by centrifugation. Pelleted material was washed in 80% ethanol and resuspended in nuclease-free water. This RNA-containing mixture was treated with DNase (Turbo DNA-*free*, Ambion), and then precipitated using ammonium acetate and ethanol. RNA quantity was measured spectrophotometrically, and quality was assessed using an Agilent Bioanalyzer.

### Microarray analysis

Custom Agilent *Francisella *microarrays designed using the eArray framework were used in this study. All predicted open reading frames, including pseudogenes, were included for *F. tularensis *subsp. *holarctica *LVS, *F. tularensis *subsp. *tularensis *(SchuS4), *F. tularensis *subsp. *holarctica *OSU18, *F. novicida *U112 and the *Francisella *plasmids, pOM1, and pFNL10. Individual genes from LVS, SchuS4, and OSU18 were spotted in duplicate on this array, whereas genes from the other strains and plasmids were printed as single copies. cDNA was synthesized from RNA by reactions using random hexamers (Invitrogen) and MMLV (Agilent). The cDNA target was labelled with Alexa Fluor 555 conjugated dUTP according to manufacturer's protocol (AF555-aha-dUTP; Invitrogen). Labelling reactions were incubated at 40°C for 3 hours. After cDNA synthesis, RNA was hydrolyzed with with NaOH and EDTA, and subsequently, the pH was neutralized with an equivalent volume of HCl. The labelled cDNA was precipitated using isopropanol and ammonium acetate and washed with 80% ethanol. 0.5 μg of labelled cDNA was hybridized to custom Agilent 8 × 15 K microarrays according to the protocol of the manufacturer and incubated at 60°C for 18 hours in a rotary oven. Following hybridization, arrays were washed with Agilent wash buffers before being scanned on an Agilent microarray scanner. At least 3 separate RNA extractions were used for each condition tested by microarray (26°C or 37°C-grown bacteria).

Gene Expression Data Analyzer (GEDA; ) was utilized for analysis of the microarray data [31]. This online software package was used for minimum mean ratio array normalization followed by log_2 _transformation. Data were grouped into two categories, cultivated at 26°C and shifted to 37°C, and analyzed for significance with the J5 statistical test [31]. The J5 metric was designed for data sets composed of limited replicates, thereby reducing the chance of generating false positives [31]. Genes were considered to be significantly differentially regulated if they had a J5 score greater than 2. Additional files 1 and 2 consist of lists showing both the J5 value and fold change for genes that were significantly up- or down-regulated upon a shift to mammalian temperature. Normalized, log_2 _transformed intensity values for genes that were differentially expressed were input into GenePattern [52] and data are presented after hierarchical clustering (Pearson correlation).

Categories assigned for clusters of orthologous groups were identified for each significantly up- or down-regulated gene based on the annotation assigned from the *F. tularensis holarctica *genome (accession, NC_07880). Specific clusters of orthologous groups categories were consolidated into general categories as follows: J, K, L, and RNA genes were combined into "Transcription/translation/DNA replication/RNA"; P, C, G, E, F, H, I, and Q were merged into "Transport and metabolism"; categories N and U were combined into "Cellular functions/trafficking/secretion"; and categories R, S, and uncategorized genes were classified as "Unknown". All other clusters of orthologous groups categories were reported as they were originally assigned.

### Quantitative real time PCR

One μg of total RNA was used as a template for cDNA synthesis catalyzed by Superscript III (Invitrogen). Diluted cDNA was used as a template for real time reactions containing primer sets (designed by Primer 3 [53] for the desired genes) and SYBR Green Supermix (BioRad). These reactions were carried out on a BioRad IQ5 real time machine. The bacterial 50S ribosomal protein L24 (FTL_0247) was used as an internal reference, as it was observed to have no change in expression following a shift to 37°C according to the microarray analysis (data not shown). The quantitative real time PCR data are presented as log_2 _transformed fold change values (37°C versus 26°C).

### Construction of plasmids

Plasmids and primers used in this study can be found in Table 1. The disruption plasmids, pMQ131hyg1581d and pMQ131hyg1664d were constructed using the following procedures. First, the PvuI/SpeI fragment of pMP615 containing *hyg *under the control of the *groEL *promoter was subcloned into pMQ131 (R. Shanks and G. O'Toole, unpublished data) that had been digested with PvuI/XbaI which produced pMQ131hyg. The internal 560 base pairs of FTL_1581 were amplified by PCR using the primers 1581_560F and 1581_560R and this amplicon was initially TA cloned into pGEM-T (Promega). The KpnI/BamHI fragment of this construct containing the internal portion of FTL_1581 was subcloned into pMQ131hyg that had been digested with these same enzymes, which produced pMQ131hyg1581d. Similarly, the internal 900 base pairs of FTL_1664 were amplified by PCR using the primers 1664_900F and 1664_900R and this amplicon was initially TA-cloned into pGEM-T (Promega). Subsequently, this fragment internal to FLT_1664 was subcloned (KpnI/BamHI) into pMQ131hyg, producing the plasmid pMQ131hyg1664d.

The FTL_1581 complementing plasmid (pFTL_1581) was constructed using the following procedures. The primers 1581_clone_up and 1581_clone_down were used to amplify 600 base pairs upstream, the entire FTL_1581 open reading frame, and 100 base pairs downstream of this gene by PCR. This amplicon encompassed the predicted native FTL_1581 promoter, this gene's coding region, as well as the predicted transcriptional stop (data not shown). Initially, this amplicon was TA-cloned into pGEM-T (Promega). The SacI/SacII fragment of this construct containing FTL_1581 was subsequently subcloned into pMQ2 (R. Shanks and G. O'Toole, unpublished data) that had been digested with these same enzymes. This yielded pFTL_1581.

The FTL_1664 (*deoB*) complementing plasmid (pFTL_1664) was constructed using the following procedures. Initially, the Kan^R ^gene from pFNLTP8 was deleted using an inverse PCR strategy [54]. The primers F8AgeI and F8XhoI were used to amplify pFNLTP8. Following PCR, template DNA was digested with DpnI leaving only amplified product. This amplicon was self-ligated, producing pF8AX. The *cat194 *gene was amplified by PCR using the primers XhoICAT and AgeICAT with pMQ2 as a template, and this amplicon was TA-cloned into pGEM-T (Promega). The Age I/XhoI fragment of this construct that encoded the *cat194 *gene was subcloned into pF8AX using these same enzymes producing pF8CAT. 1664_clone_up and 1664_clone_down were used to amplify 600 base pairs upstream, the entire FTL_1664 open reading frame, and 100 base pairs downstream of this gene by PCR. This amplicon encompassed the predicted native FTL_1664 promoter, this gene's coding region, as well as the predicted transcriptional stop (data not shown). Initially, this amplicon was TA-cloned into pGEM-T(Promega). The BamHI/KpnI fragment of this construct containing FTL_1664 was subsequently subcloned into pF8CAT that had been digested with these same enzymes. This yielded pFTL_1664.

### Construction of the FTL_1664 and FTL_1581 mutants

*F. 0tularensis *LVS disruption mutants of FTL_1664 and FTL_1581 were constructed as previously reported [55]. A homologous recombination between the LVS chromosomal copy of FTL_1581 and the internal FTL_1581 fragment in pMQ131hyg1581d would disrupt the coding sequence of 38 amino acids at the C-terminus. Also, homologous recombination between chromosomal FTL_1664 and pMQ131hyg1664d would disrupt coding sequence for the C-terminal 56 amino acids of the encoded protein. Disruption constructs (either pMQ131hyg1664d or pMQ131hyg1581d) were mobilized into *F. tularensis *LVS by triparental mating. Mating mixes were composed of LVS (10^9 ^CFU), *E. coli*/pRK2013 (10^7 ^CFU), and either *E. coli*/pMQ131hyg1664d or *E. coli*/pMQ131hyg1581d (10^7 ^CFU). Similarly to the previously described *Francisella *conjugation methodology [56], the mating mixture here was plated on LB agar plates, and incubated at room temperature for 18 hours. Cells were resuspended in phosphate buffered saline (PBS) and spread onto chocolate II agar plates containing polymyxin (100 μg/ml) and hygromycin. Hygromycin-resistant colonies were screened by PCR using primers specific for either FTL_1581 or FTL_1664 and the vector-portion of pMQ131hyg1581d or pMQ131hyg1664d. The *F. tularensis *LVS FTL_1664 and FTL_1581 disruption mutants (FTL_1664::pMQ131hyg1664d; FTL_1581::pMQ131hyg1581d) are referred to as strains 1664d and 1581d respectively. Mobilization of complementation constructs into the disruption mutants was accomplished by electroporation as previously described [57].

### Complement resistance assay

Bacteria (1.5 × 10^9 ^CFU) were incubated in solutions of PBS with 20% serum (human serum [Gemini Bioscience]) or 20% heat-inactivated serum at 37°C while shaking at 250 rpm. Serum was incubated at 56°C for 30 minutes to inactivate complement. At certain time-points, cell suspensions were diluted and plated to enumerate CFU.

### Chicken embryo infections

Chicken embryos were infected with *F. tularensis *LVS, 1664d, and 1581d as previously described [37,38]. Whiteleghorn chicken eggs (fertilized, specific pathogen free) were purchased from Charles River Laboratories, North Franklin, CT, USA. Eggs were incubated at 37°C with gentle rocking and humidity for one week prior to infection. Following the initial 7 day incubation, those without a live embryo were discarded. For infections, the surface of the egg shell was sterilized with 70% ethanol. The egg shell membrane was exposed and removed after introducing a small hole in the shell. Bacteria suspended in PBS (100 μl) were then injected beneath the chorioallantoic membrane. The hole in the egg shell was covered with transparent tape and subsequently eggs were incubated as previously described. On a daily basis, eggs were candled to assess viability for 6 days. As previously reported [37], embryos that expired within 24 h of the infection were presumed to have experienced lethal trauma during inoculation, and were removed from the experiment. Survival differences in chicken embryo infections were analyzed with the log rank test in GraphPad Prism 5.

Competition studies in the chicken embryo infection model were conducted similar to the single infections described above. Here chicken embryos were infected with a mixture of LVS and 1581d (1:1 based on OD_600_, ~10^5 ^total bacteria). Actual input (6.5 × 10^5 ^total bacteria) was determined by diluting the inoculum followed by plating to enumerate viable bacteria. Three infected, viable eggs were sacrificed at 1, 2, and 3 days (9 total eggs) post infection. Egg contents were homogenized with an OMNI tissue homogenizer, and serial dilutions of the homogenates were plated on chocolate II agar plates with or without antibiotic. The total number of 1581d (plates with antibiotic) was subtracted from the number of total bacteria (plates without antibiotic) to determine the amount of viable wild-type LVS. Competition ratios (1581d: LVS) were normalized to the input to account for small differences in the inocula, as has been done previously [58,59]. Competition ratios were analyzed for statistical significance by Chi square.

### Mammalian cell infections

Human monocytes were differentiated into macrophages by *in vitro *culture as has been described previously [11,57,60]. Buffy coats from blood donations (Central Blood Bank, Pittsburgh, Pennsylvania) served as the source of monocytes. Monocytes were purified using Ficoll gradients (Amersham Biosciences) to isolate PBMCs, Optiprep gradients (Axis-Shield) to enrich for monocytes, and panning on plastic to further purify monocytes (final purity >95% based on microscopy). Cells were cultured in 60 mm culture dishes for 7 days at 37°C with 5% CO_2 _in 7 ml of DMEM (Invitrogen) supplemented with 20% FCS (Invitrogen), 10% human serum (Gemini Biosciences), 25 mM HEPES (Gibco), and 1% Glutamax (Invitrogen) [11,57,60]. Macrophages were detached from the culture dish on day 7 using a lidocaine/EDTA solution (5 mM EDTA and 4 mg/ml lidocaine in PBS pH 7.2). For monocyte-derived dendritic cells, human monocytes were seeded at a density of 1 × 10^6 ^cells/ml in 24 well plates (Costar) in complete RPMI [10% FCS, 25 mM HEPES, 1% non-essential amino acids, 1% sodium pyruvate, 1% Glutamax and 0.1% 2-mercaptoethanol (all from Gibco)] supplemented with 1000 U/ml GM-CSF and 1000 U/ml IL-4 (both from eBioscience) and incubated at 37°C with 5% CO_2_. On day 3 of culture, 10% of the media was replaced with fresh complete RPMI supplemented with GM-CSF and IL-4 and non-adherent cells were harvested at day 5. For *in vitro *infections, all primary cells were washed and resuspended in DMEM supplemented with 1% human serum, 25 mM HEPES, and 1% Glutamax and then plated onto Primaria-coated 96-well culture dishes (Becton, Dickinson and Company) at a density of 5.0 × 10^4 ^cells per well.

Murine macrophage-like RAW 264.7 cells (ATCC number TIB-71) were routinely cultured in DMEM supplemented with 10% fetal calf serum, 25 mM HEPES, and 1% Glutamax with 100 U/ml penicillin-streptomycin. Two days prior to infection, the medium for these cells was replaced with DMEM supplemented with 10% human serum, 25 mM HEPES, and 1% Glutamax, and infections were carried out using this same medium.

HEK-293 cells (ATCC number CRL-1573), a non-phagocytic cell line [61], were cultivated in DMEM supplemented with 10% fetal calf serum, 25 mM HEPES, and 1% Glutamax with 100 U/ml penicillin-streptomycin. One day prior to infection, HEK-293 cells were seeded in 96-well plates at a concentration of 5 × 10^4 ^cells per well in this same medium devoid of antibiotic and infections were carried out in this same medium.

For infection experiments, bacterial cultures were adjusted to an OD_600 _of 0.3 (approximately 1.5 × 10^9 ^CFU/ml) and diluted to attain a multiplicity of infection (MOI) of 500, which typically yields infection rate >80% after a 2 hour co-incubation [11,62]. Alternatively, macrophages were suspended in DMEM supplemented with 10% human serum, 25 mM HEPES, and 1% Glutamax and transferred to culture dishes before infection. Prior to administration of the bacteria onto the macrophages, the *F. tularensis *strains were incubated at 37°C with 5% CO_2 _for 20 min in this same medium. Macrophages were infected with bacteria that had been diluted to an MOI of 40. This alternative infection protocol used a lower MOI because it took advantage of the complement-mediated uptake of *F. tularensis *into macrophages [47]. Comparable results were obtained regardless of which infection protocol was used. Actual MOIs were measured by plating serial dilutions of inocula on chocolate II agar plates.

For intracellular CFU enumeration, mammalian cells were exposed to the initial bacterial load for 2 hours at 37°C with 5% CO_2 _and then incubated with gentamicin (20 μg/ml) for 20 min to kill extracellular bacteria. The cells were washed with warm Hank's balanced salt solution and then incubated at 37°C with 5% CO_2 _for another 22–48 h with fresh culture media. At the indicated time-points post-infection, mammalian cells were lysed with 0.02% sodium dodecyl sulfate. The 2 hour time point was used to determine entry similarly to a prior study [46]. Serial dilutions of the lysates were plated on chocolate II agar plates for enumeration of viable bacteria. CFU counts were converted to log_10 _values and analyzed by the Student's t-Test to determine statistical differences. For uptake analysis, CFU were normalized to actual MOI to account for minor variations in the density of inocula [46].

For fluorescence microscopy, bacteria were stained with 10 μM Syto 9 (Invitrogen) for 15 min and washed three times with PBS prior to infection. Here, stained cells were diluted in DMEM supplemented with 10% human serum, 25 mM HEPES, and 1% Glutamax, and were incubated at 37°C with 5% CO_2 _for 20 min. These bacteria were used to inoculate RAW 264.7 in this same medium at an MOI of 10.

## Authors' contributions

JH wrote the manuscript, constructed plasmids and mutants, conducted the experiments, envisioned and designed the study. PEC designed the microarrays and contributed toward the execution of microarray experiments. DMO conducted the real time PCR. Both PEC and DMO assisted with the chicken embryo infections. RMQS constructed plasmids and participated in the drafting of the manuscript. GJN oversaw the work, contributed to the conception and design of this research, and participated in the drafting of this manuscript. All authors read and approved the final version of the manuscript.

## Supplementary Material

Additional file 1 **Table S1.** This table shows a list of *F. tularensis *LVS genes significantly induced at 37°C. Click here for file

Additional file 2 **Table S2.** This table displays a list of *F. tularensis *LVS genes significantly down-regulated at 37°C. Click here for file
