# Supplementary material for: Global transcriptional response to mammalian temperature provides new insight into Francisella tularensis pathogenesis
Source: BMC Microbiol. 2008 Oct 8;8:172. doi: 10.1186/1471-2180-8-172 (PMC2576331; doi:10.1186/1471-2180-8-172)
Supplement: Additional file 1 — Table S1. This table shows a list of F. tularensis LVS genes significantly induced at 37°C. [file 1471-2180-8-172-S1.doc]

Table S1. *F. tularensis* LVS genes significantly induced at 37ºC.

| FTL_# | Annotation | J5 | Fold Change |
| --- | --- | --- | --- |
| FTL_R0032 | 5S ribosomal RNA | 6.6 | 13.8 |
| FTL_1715 | Chaperonin protein, *groES* | 4.9 | 3.7 |
| FTL_R0042 | tRNA-Met | 4.2 | 4.5 |
| FTL_1714 | Chaperone protein, *groEL* | 4.0 | 3.2 |
| FTL_R0012 | tRNA-Arg | 3.8 | 3.4 |
| FTL_0029 | Carbamoyl-phosphate synthase large chain | 3.8 | 2.6 |
| FTL_1595 | dimethyladenosine transferase , kasugamycin resistance | 3.8 | 3.2 |
| FTL_R0023 | tRNA-Asn | 3.6 | 5.1 |
| FTL_1047 | 30S ribosomal protein S21 | 3.5 | 3.1 |
| FTL_1581 | hypothetical lipoprotein | 3.4 | 3.1 |
| FTL_R0046 | tRNA-Thr | 3.3 | 2.3 |
| FTL_0030 | Carbamoyl-phosphate synthase small chain | 3.3 | 2.6 |
| FTL_1957 | heat shock protein | 3.3 | 2.6 |
| FTL_0887 | o-methyltransferase family protein | 3.2 | 2.8 |
| FTL_0713 | PP-loop family protein | 3.2 | 2.6 |
| FTL_0307 | Dephospho-CoA kinase | 3.1 | 2.1 |
| FTL_0930 | holliday junction endodeoxyribonuclease | 3.1 | 2.7 |
| FTL_R0036 | tRNA-His | 2.9 | 2.6 |
| FTL_1545 | SNO glutamine amidotransferase family protein | 2.9 | 2.6 |
| FTL_0094 | ClpB protein | 2.8 | 2.5 |
| FTL_R0038 | tRNA-Pro | 2.8 | 2.1 |
| FTL_0558 | Oxidoreductase | 2.8 | 2.2 |
| FTL_0337 | pseudogene | 2.8 | 2.0 |
| FTL_0267 | Chaperone Hsp90, heat shock protein HtpG | 2.8 | 2.3 |
| FTL_0225 | protein chain elongation factor EF-Ts | 2.8 | 2.0 |
| FTL_0610 | transcription termination factor Rho | 2.8 | 2.4 |
| FTL_0445 | hypothetical protein | 2.8 | 2.5 |
| FTL_1433 | arabinose phosphate isomerase | 2.8 | 2.3 |
| FTL_R0051 | tRNA-Asp | 2.8 | 3.9 |
| FTL_0028 | aspartate carbamoyltransferase | 2.8 | 2.2 |
| FTL_1794 | ATP synthase epsilon chain | 2.7 | 2.1 |
| FTL_0671 | Annotated as transcriptional regulator, homologous to Pantothenate kinase type III, *coaX* | 2.7 | 2.3 |
| FTL_0345 | bile acid symporter family protein | 2.7 | 2.2 |
| FTL_R0037 | tRNA-Arg | 2.6 | 2.0 |
| FTL_1362 | hypothetical protein | 2.6 | 2.2 |
| FTL_0929 | hypothetical protein | 2.6 | 2.4 |
| FTL_0848 | preprotein translocase, subunit D, membrane protein | 2.6 | 2.1 |
| FTL_1546 | Pyridoxine/pyridoxal 5-phosphate biosynthesis protein | 2.6 | 2.2 |
| FTL_0675 | hypothetical protein | 2.6 | 2.3 |
| FTL_0828 | Type IV pili nucleotide binding protein, ABC transporter, ATP-binding protein | 2.6 | 2.2 |
| FTL_0684 | hypothetical lipoprotein | 2.5 | 2.3 |
| FTL_1795 | ATP synthase beta chain | 2.5 | 1.9 |
| FTL_0886 | conserved hypothetical protein *yleA* | 2.5 | 1.8 |
| FTL_1485 | hypothetical protein | 2.5 | 2.2 |
| FTL_0672 | Aspartate-1-decarboxylase | 2.5 | 2.0 |
| FTL_R0047 | tRNA-Gly | 2.5 | 1.7 |
| FTL_1044 | hypothetical protein | 2.5 | 2.2 |
| FTL_R0033 | tRNA-Val | 2.4 | 2.0 |
| FTL_1239 | signal recognition particle protein, Ffh | 2.4 | 1.8 |
| FTL_R0011 | tRNA-Gly | 2.4 | 2.6 |
| FTL_0648 | Aminotransferase | 2.4 | 2.2 |
| FTL_1784 | 2-oxoglutarate dehydrogenase E1 component | 2.4 | 1.8 |
| FTL_0731 | YhhQ family protein | 2.4 | 2.3 |
| FTL_1139 | 3-oxoacyl-(acyl-carrier-protein) reductase | 2.4 | 1.8 |
| FTL_1191 | Chaperone protein dnaK (heat shock protein family 70 protein) | 2.4 | 1.8 |
| FTL_1664 | Phosphopentomutase | 2.4 | 2.1 |
| FTL_1743 | DNA-directed RNA polymerase, beta subunit | 2.4 | 1.4 |
| FTL_0837 | D-methionine binding transport protein, ABC transporter, membrane and periplasmic protein | 2.4 | 2.0 |
| FTL_1190 | Chaperone protein grpE (heat shock protein family 70 cofactor) | 2.4 | 1.7 |
| FTL_1021 | hypothetical protein | 2.4 | 2.0 |
| FTL_1338 | alanine racemase | 2.4 | 2.1 |
| FTL_0849 | preprotein translocase, subunit F, membrane protein | 2.3 | 2.1 |
| FTL_1108 | cytosol aminopeptidase family protein | 2.3 | 2.2 |
| FTL_1043 | hypothetical protein | 2.3 | 2.0 |
| FTL_0964 | ATP-dependent protease, ATP-binding subunit | 2.3 | 1.8 |
| FTL_1474 | transcriptional elongation factor | 2.3 | 1.8 |
| FTL_1302 | phenol hydroxylase | 2.3 | 2.1 |
| FTL_0309 | pyruvate dehydrogenase, E1 component | 2.3 | 1.8 |
| FTL_1067 | hypothetical protein | 2.3 | 2.1 |
| FTL_0210 | Valyl-tRNA synthetase | 2.2 | 2.0 |
| FTL_0928 | DJ-1/PfpI family protein | 2.2 | 2.1 |
| FTL_0673 | Pantoate-beta-alanine ligase | 2.2 | 1.8 |
| FTL_1796 | ATP synthase gamma chain | 2.2 | 1.4 |
| FTL_1140 | malonyl coA-acyl carrier protein transacylase | 2.2 | 1.8 |
| FTL_1935 | ABC transporter, ATP-binding protein | 2.2 | 1.7 |
| FTL_0198 | Pyridoxal/pyridoxine/pyridoxamine kinase | 2.2 | 2.0 |
| FTL_1137 | 3-oxoacyl-[acyl-carrier-protein] synthase II | 2.2 | 1.9 |
| FTL_0885 | phoH-like protein | 2.2 | 1.9 |
| FTL_0954 | hypothetical lipoprotein | 2.2 | 2.0 |
| FTL_1232 | potassium uptake protein TrkA | 2.2 | 1.8 |
| FTL_R0014 | tRNA-Ser | 2.1 | 3.2 |
| FTL_0280 | amino acid permease, fragment | 2.1 | 1.7 |
| FTL_1147 | phosphoglycerate kinase | 2.1 | 1.8 |
| FTL_R0009 | tRNA-Glu | 2.1 | 2.9 |
| FTL_0743 | Oxidoreductase, short-chain dehydrogenase family protein | 2.1 | 1.9 |
| FTL_0899 | protease, GTP-binding subunit | 2.1 | 1.8 |
| FTL_1842 | Glutamyl-tRNA(Gln) amidotransferase subunit A | 2.1 | 1.8 |
| FTL_1553 | Succinyl-CoA synthetase beta chain | 2.1 | 1.6 |
| FTL_1311 | CTP synthase | 2.1 | 1.9 |
| FTL_1068 | tRNA pseudouridine synthase A | 2.1 | 1.9 |
| FTL_0548 | HAM1 protein | 2.0 | 1.8 |
| FTL_1797 | ATP synthase alpha chain | 2.0 | 1.6 |
| FTL_1048 | hypothetical protein | 2.0 | 1.8 |
| FTL_1782 | adenine phosphoribosyltransferase | 2.0 | 1.5 |
| FTL_0479 | glycine cleavage system P protein, subunit 1 | 2.0 | 1.4 |
