# Supplementary material for: Global transcriptional response to mammalian temperature provides new insight into Francisella tularensis pathogenesis
Source: BMC Microbiol. 2008 Oct 8;8:172. doi: 10.1186/1471-2180-8-172 (PMC2576331; doi:10.1186/1471-2180-8-172)
Supplement: Additional file 2 — Table S2. This table displays a list of F. tularensis LVS genes significantly down-regulated at 37°C. [file 1471-2180-8-172-S2.doc]

Table S2. *F. tularensis* LVS genes significantly down-regulated at 37ºC.

| FTL_# | Annotation | J5 | Fold Change |
| --- | --- | --- | --- |
| FTL_0158 | acid phosphatase (precursor) | -4.2 | -4.0 |
| FTL_1560 | pseudogene | -4.2 | -2.8 |
| FTL_1719 | hypothetical protein | -4.1 | -3.8 |
| FTL_1846 | hypothetical protein | -4.1 | -4.1 |
| FTL_1104 | pseudogene | -4.1 | -2.9 |
| FTL_1083 | hypothetical protein | -4.1 | -4.4 |
| FTL_1360 | 30S ribosomal protein S21 | -4.0 | -3.9 |
| FTL_1768 | pseudogene | -4.0 | -2.8 |
| FTL_1697 | metal ion transporter | -3.9 | -3.0 |
| FTL_1675 | pseudogene | -3.9 | -3.9 |
| FTL_1361 | cold shock protein | -3.9 | -3.2 |
| FTL_0471 | hypothetical protein | -3.9 | -2.0 |
| FTL_0456 | 30S ribosomal protein S21 | -3.9 | -3.6 |
| FTL_1712 | HsdR protein, fragment | -3.8 | -3.9 |
| FTL_1928 | pseudogene | -3.8 | -3.3 |
| FTL_1698 | hypothetical protein | -3.7 | -3.0 |
| FTL_1884 | hypothetical protein | -3.5 | -3.2 |
| FTL_0457 | cold shock protein | -3.5 | -3.2 |
| FTL_1677 | pseudogene | -3.5 | -3.5 |
| FTL_1839 | Lipase/acyltransferase | -3.4 | -2.1 |
| FTL_0446 | hypothetical protein | -3.4 | -2.9 |
| FTL_1052 | putative glycosidase | -3.4 | -2.9 |
| FTL_0774 | pseudogene | -3.4 | -4.1 |
| FTL_0039 | hypothetical membrane protein | -3.3 | -3.1 |
| FTL_0064 | pseudogene | -3.3 | -2.2 |
| FTL_1920 | pseudogene | -3.2 | -3.2 |
| FTL_0067 | pseudogene | -3.2 | -3.2 |
| FTL_0772 | pseudogene | -3.2 | -2.1 |
| FTL_1730 | hypothetical protein | -3.2 | -2.9 |
| FTL_0991 | pseudogene | -3.2 | -1.4 |
| FTL_1515 | ABC transporter, membrane protein | -3.1 | -3.1 |
| FTL_0936 | hypothetical protein | -3.1 | -2.7 |
| FTL_0467 | hypothetical protein | -3.1 | -2.6 |
| FTL_1653 | peptide transport system substrate-binding protein | -3.1 | -2.6 |
| FTL_1456 | pseudogene | -3.1 | -2.7 |
| FTL_0303 | hypothetical protein | -3.0 | -2.6 |
| FTL_1890 | pseudogene | -3.0 | -2.5 |
| FTL_1260 | pseudogene | -3.0 | -2.4 |
| FTL_1367 | hypothetical protein | -3.0 | -2.5 |
| FTL_0557 | Delta 9 acyl-lipid fatty acid desaturase | -3.0 | -2.9 |
| FTL_1573 | major facilitator superfamily (MFS) transport protein | -2.9 | -2.5 |
| FTL_0038 | HlyD family secretion protein | -2.9 | -2.3 |
| FTL_1711 | HsdR protein, fragment | -2.8 | -2.3 |
| FTL_0869 | hypothetical protein | -2.8 | -2.3 |
| FTL_1676 | pseudogene | -2.8 | -2.3 |
| FTL_0442 | hypothetical protein | -2.8 | -2.6 |
| FTL_1128 | hypothetical protein | -2.8 | -2.6 |
| FTL_1221 | hypothetical membrane protein | -2.7 | -2.5 |
| FTL_1951 | Transporter, LysE family | -2.7 | -2.7 |
| FTL_1219 | Aminotransferase, class II | -2.7 | -2.6 |
| FTL_0021 | Proline/betaine transporter, major facilitator superfamily (MFS) transport protein | -2.7 | -2.4 |
| FTL_1084 | hypothetical membrane protein | -2.7 | -2.7 |
| FTL_0041 | hypothetical protein | -2.7 | -2.4 |
| FTL_0055 | hypothetical protein | -2.7 | -2.1 |
| FTL_1919 | pseudogene | -2.7 | -2.2 |
| FTL_0066 | pseudogene | -2.7 | -2.7 |
| FTL_0037 | hypothetical membrane protein | -2.7 | -2.2 |
| FTL_0734 | pseudogene | -2.7 | -2.5 |
| FTL_0313 | hypothetical protein | -2.6 | -2.8 |
| FTL_0819 | pseudogene | -2.6 | -2.0 |
| FTL_0495 | pseudogene | -2.6 | -1.9 |
| FTL_0925 | Proton-dependent oligopeptide transport (POT) family protein | -2.6 | -2.3 |
| FTL_1518 | pseudogene | -2.6 | -2.3 |
| FTL_1918 | ribosomal protein S6 modification protein-related protein | -2.6 | -2.2 |
| FTL_1651 | pseudogene | -2.6 | -2.4 |
| FTL_0635 | FAD-dependent pyridine nucleotide-disulphide oxidoreductase | -2.5 | -2.3 |
| FTL_0511 | hypothetical protein | -2.5 | -2.6 |
| FTL_1183 | Sodium-solute symporter family protein | -2.5 | -2.4 |
| FTL_1315 | hypothetical protein | -2.5 | -3.0 |
| FTL_0730 | haloacid dehalogenase-like hydrolase family protein | -2.5 | -2.3 |
| FTL_0497 | pseudogene | -2.5 | -2.2 |
| FTL_1561 | pseudogene | -2.5 | -2.1 |
| FTL_0052 | hypothetical protein | -2.5 | -2.3 |
| FTL_0634 | NADH oxidase | -2.4 | -2.5 |
| FTL_0163 | MFS transporter | -2.4 | -2.2 |
| FTL_1176 | LysR transcriptional regulator family protein | -2.4 | -2.3 |
| FTL_1588 | mechanosensitive ion channel protein | -2.4 | -2.2 |
| FTL_1270 | pseudogene | -2.4 | -2.1 |
| FTL_0003 | conserved hypothetical membrane protein, fragment | -2.4 | -2.2 |
| FTL_1758 | hypothetical protein | -2.4 | -2.3 |
| FTL_0818 | pseudogen | -2.4 | -1.8 |
| FTL_1761 | hypothetical protein | -2.4 | -2.1 |
| FTL_1887 | 3-isopropylmalate dehydrogenase | -2.3 | -2.1 |
| FTL_1457 | pseudogene | -2.3 | -1.9 |
| FTL_1256 | pseudogene | -2.3 | -2.4 |
| FTL_1154 | pseudogene | -2.3 | -2.3 |
| FTL_1435 | hypothetical protein | -2.3 | -2.0 |
| FTL_0417 | Transposase | -2.3 | -2.2 |
| FTL_1729 | (Di)nucleoside polyphosphate hydrolase | -2.3 | -2.3 |
| FTL_1652 | pseudogene | -2.3 | -2.1 |
| FTL_0053 | Beta-fructofuranosidase | -2.2 | -1.9 |
| FTL_1395 | Galactose-proton symporter, major facilitator superfamily (MFS) transport protein | -2.2 | -2.5 |
| FTL_1886 | Transposase | -2.2 | -2.2 |
| FTL_0342 | deoxyribodipyrimidine photolyase | -2.2 | -2.1 |
| FTL_1691 | hypothetical protein | -2.2 | -1.8 |
| FTL_1765 | cytochrome oxidase bd-II, subunit II | -2.2 | -2.2 |
| FTL_0564 | pseudogene | -2.2 | -1.8 |
| FTL_0992 | pseudogene | -2.2 | -2.2 |
| FTL_1318 | pseudogene | -2.2 | -2.0 |
| FTL_1092 | pseudogene | -2.2 | -2.1 |
| FTL_1472 | hypothetical protein | -2.2 | -2.1 |
| FTL_1152 | pseudogene | -2.2 | -1.7 |
| FTL_1517 | hypothetical protein | -2.2 | -1.9 |
| FTL_1844 | Secretion protein | -2.2 | -2.2 |
| FTL_1056 | pseudogene | -2.2 | -2.0 |
| FTL_1681 | Transposase | -2.2 | -2.1 |
| FTL_0263 | Transposase | -2.2 | -2.2 |
| FTL_0034 | hypothetical protein | -2.2 | -2.0 |
| FTL_0385 | pseudogene | -2.1 | -2.1 |
| FTL_0952 | hypothetical protein | -2.1 | -2.1 |
| FTL_1299 | hypothetical protein | -2.1 | -2.0 |
| FTL_1944 | Transposase | -2.1 | -2.1 |
| FTL_1439 | Arylsulfatase | -2.1 | -2.8 |
| FTL_0817 | pseudogene | -2.1 | -1.9 |
| FTL_1894 | Transposase, fragment | -2.1 | -1.9 |
| FTL_R0031 | 23S ribosomal RNA | -2.1 | -2.1 |
| FTL_1440 | metal-dependent hydrolase, beta-lactamase superfamily III | -2.1 | -1.7 |
| FTL_1209 | hypothetical protein | -2.1 | -1.9 |
| FTL_1054 | putative alpha-xylosidase | -2.1 | -2.0 |
| FTL_0264 | pseudogene | -2.1 | -1.9 |
| FTL_1091 | pseudogene | -2.0 | -2.1 |
| FTL_1103 | pseudogene | -2.0 | -1.9 |
| FTL_1195 | Transposase | -2.0 | -2.1 |
| FTL_0386 | pseudogene | -2.0 | -2.0 |
| FTL_0759 | hypothetical membrane protein | -2.0 | -2.0 |
